# P372: Reduction of HAI Legionella pneumophilia pneumonia and Pseudomonas aeruginosa sepsis by control with water supply

**DOI:** 10.1186/2047-2994-2-S1-P372

**Published:** 2013-06-20

**Authors:** LP Andersen, A-MB Hellesoe, B Rubenhagen, M Høg

**Affiliations:** 1 Infection Control 9101, Copenhagen University Hospital, Rigshospitalet, Copenhagen, Denmark; 2 Clinical Microbiology, Copenhagen University Hospital, Rigshospitalet, Copenhagen, Denmark

## Introduction

Water associated hospital acquired infections with *Legionella pneumophila* and *Pseudomonas aeruginosa* is a problem in many Hospital settings. At Rigshospitalet there was a high incidence of HAI *L. pneumophilia* pneumonia compared to other Danish hospitals. I addition several dialyze patients with *P. aeruginosa* septicemia could only be explained by infection through the dialyze catheter during showers.

## Objectives

Is it possible to reduce the number of water associated HAI by systematical control of water supply and focused infection control precautions in wards at risk?

## Methods

Total germ count and *Legionella* germ count was measured in shower water and drinking water twice a year. Laboratory and standardized in vivo tests were done on shower water. Incidence rates of HAI *L. pneumophilia* pneumonia, *P. aeruginosa*, *Acinetobacter baumanii* and *Stenotrophamonas maltophilia* septicemia in hospitalized patients were recorded.

## Results

Both the incidence rates of HAI *L. pneumophilia* pneumonia, *P. aeruginosa* septicemia in hospitalized patients were reduced more than 50% within a few years.

Figures cannot be shown.

## Conclusion

Systematical control of water supply and focused infection control precautions in wards at risk can reduce the number of water associated HAI over time.

## Disclosure of interest

None declared

